# Gene Therapy Targeting HIV Entry

**DOI:** 10.3390/v6031395

**Published:** 2014-03-21

**Authors:** Chuka Didigu, Robert Doms

**Affiliations:** 1 Department of Microbiology, Perelman School of Medicine, University of Pennsylvania, 3610 Hamilton Walk, Philadelphia, PA 19104, USA; E-Mail: cdidigu@mail.med.upenn.edu; 2 Department of Pathology, Children’s Hospital of Philadelphia, 34th and Civic Center Blvd-5th Floor Main Building, Philadelphia, PA 19104, USA

**Keywords:** HIV, gene therapy, HIV cure, HIV entry, HIV reservoir, CCR5 delta 32

## Abstract

Despite the unquestionable success of antiretroviral therapy (ART) in the treatment of HIV infection, the cost, need for daily adherence, and HIV-associated morbidities that persist despite ART all underscore the need to develop a cure for HIV. The cure achieved following an allogeneic hematopoietic stem cell transplant (HSCT) using HIV-resistant cells, and more recently, the report of short-term but sustained, ART-free control of HIV replication following allogeneic HSCT, using HIV susceptible cells, have served to both reignite interest in HIV cure research, and suggest potential mechanisms for a cure. In this review, we highlight some of the obstacles facing HIV cure research today, and explore the roles of gene therapy targeting HIV entry, and allogeneic stem cell transplantation in the development of strategies to cure HIV infection.

## 1. Introduction

The introduction of antiretroviral therapy (ART) for HIV infection has dramatically altered the course of the HIV pandemic by delaying the progression to AIDS and increasing the lifespan of infected individuals [1,2,3,4], while decreasing HIV transmission rates [5,6,7,8]. However, the prospect of eradication remains a distant one, as there are still 34 million people living with HIV worldwide, and more than 2 million new infections every year. Additionally, as the HIV infected population ages, we are learning that control of HIV infection by ART does not completely restore health (reviewed in [9]). Patients on ART remain at increased risk of developing cardiovascular disease compared to age-matched uninfected controls [10], and HIV infection also significantly increases the risk of developing kidney disease, osteoporosis, and a number of non-AIDS defining malignancies, even when patients are well controlled on ART [11,12,13,14]. Moreover, ART itself is not without side effects, as certain classes of antiretroviral drugs directly contribute to some of these comorbidities. These HIV-associated morbidities that persist despite ART, the high cost of ART, and the requirement for daily adherence to ART to control infection, all highlight the pressing need for strategies to achieve drug-free control and an eventual cure for HIV. 

## 2. Barriers to a Cure: The HIV Reservoir

A major obstacle to achieving a cure stems from the fact that HIV not only infects and kills cells involved in combating infection (CD4 T cells), but also establishes a stable reservoir in these cells that goes undetected by the immune system [15,16,17,18]. This latent reservoir is present in resting memory CD4 T cells, is largely unaffected by ART, and is responsible for the rapid rebound in viremia following ART cessation [17,18,19,20,21]. As such, elimination of the reservoir is likely required for a cure. Based on our understanding of HIV latency, it has long been thought that reactivation of these latent reservoirs would result in the detection of infected cells and their subsequent destruction by the immune system. The pursuit of this “shock-and-kill” approach has led to the identification of pharmacologic compounds capable of reactivating latent HIV *in vitro* (reviewed in [22]), and there is evidence that one such drug, the histone deacetylase inhibitor vorinostat, is capable of disrupting HIV-1 latency to some degree in HIV infected individuals on ART [23]. However, a recent study by Shan *et al.* showed that following drug-induced reactivation of latency in CD4 T cells from patients on ART, these cells were not killed by autologous cytotoxic lymphocytes (CTLs), due to defects in the quality of the HIV-specific CTL response in ART-experienced patients [24]. These results suggest that pharmacologic reactivation of latency alone may not be enough to eliminate the reservoir. As such, there is a need for alternative or complementary approaches that will enhance the ability of the immune system of HIV-infected persons to identify and kill cells harboring reactivated viruses if we hope to effectively eliminate the HIV reservoir using this strategy. A recent landmark study by Ho et al has also called into question our previous estimates of the HIV reservoir, and the ease with which the reservoir can be reactivated [25]. This work stemmed from the observation that DNA measurements of the HIV reservoir size are up to 2-logs higher than measurements obtained using the standard viral outgrowth assay—an assay that estimates the reservoir size by measuring HIV production following maximum *in vitro* T cell activation of resting CD4 T cells from HIV-infected individuals [26]. This difference in reservoir size estimates was initially believed to represent defective proviruses—a belief supported by the error-prone nature of HIV replication [27,28,29,30,31,32]. However, a careful characterization of these uninduced proviruses by Ho and colleagues revealed that up to 12% of them are actually genetically intact, integrate into active sites of transcription, and when synthesized, display replication kinetics comparable to those of latent viruses induced by T cells activation. This study has 2 major implications for the shock-and-kill approach and the HIV cure field in general—the replication-competent HIV reservoir is considerably larger than previously thought, and reactivation of latent HIV is not determined solely by the activation state of a T cell, but may in fact be in part a stochastic process.

## 3. Gene Therapy for a Cure

Genetic manipulation of long-lived primary CD4 T cells and hematopoietic stem cells (HSCs) to prevent HIV infection has long been viewed as a viable means of achieving ART-free control of infection, and following the recent report of a cure for HIV [33,34], there has been a surge of interest in exploring gene therapy-based approaches to treat HIV. This cure was achieved following an allogeneic hematopoietic stem cell transplant (HSCT) to treat an HIV-infected man with leukemia using cells from a donor with an inactivating mutation in both copies of *ccr5*—the primary HIV entry coreceptor [33,34]. This mutation—known as *ccr5*∆32—confers resistance to HIV infection in homozygotes, and delays the progression to AIDS in heterozygotes [35,36,37]. Following the transplant, the patient was taken off ART and in the ensuing years has remained free of HIV, with undetectable viral loads and substantial decreases in HIV-specific antibodies, suggesting that a cure has indeed been achieved. However, there remains a great deal of speculation regarding the reason for the cure. One possible explanation is that the complete donor chimerism achieved following allogeneic transplantation of CCR5-negative cells simply created an environment incapable of supporting HIV infection by eradicating all cells susceptible to infection with CCR5-using HIV. However, this may not fully explain the observed cure as the patient in question also had low levels of HIV capable of entering cells in a CCR5-independent manner by using the CXCR4 coreceptor, and yet these viruses failed to expand following the transplant. That such viruses can expand *in vivo* in the face of selective pressure against CCR5 is evidenced by the fact that the most common cause of virologic failure following treatment with the CCR5 antagonist maraviroc is outgrowth of pre-existing CXCR4-using HIV strains [38,39]. As such, alternative explanations for the cure including the role of graft- *vs.* host-disease (GVHD) in clearing the infection have been considered, with the assumption that the development of GVHD following transplant led to the detection and donor cell-mediated clearance of all host immune cells including those cells comprising the latent HIV reservoir. Another potential reason for this remarkable cure is the destruction of the HIV reservoir by the conditioning chemotherapy and total body irradiation administered prior to the transplant. While such transplants have been performed in HIV infected patients in the past with no effect on their HIV infection, recent evidence suggests that allogeneic stem cell transplants with CCR5-positive cells may in fact have an effect on the size of the HIV reservoir as measured by the viral outgrowth assay [40].

The striking HIV-resistant phenotype observed in *ccr5*∆32 homozygotes and the recent report of a cure following HSCT using *ccr5*∆32 cells has spurred several gene therapy efforts to block HIV infection at the level of entry in an attempt to reproduce this HIV-resistant phenotype in individuals lacking the delta32 mutation. As a result, several promising preclinical studies have successfully provided some form of protection against HIV by targeting infection at the level of entry and a number of these studies have been advanced to human clinical trials (Table 1). 

**Table 1:** Gene therapy clinical trials targeting the major steps in HIV entry.

Steps in entry targeted	Transgene/Payload	Target cell	% gene marked cells at study conclusion	Ref.
CD4 binding	CD4-zeta chimeric T cell receptor	Autologous CD4^+^ and CD8^+^ T cells	0.1% of PBMCs at 1 year	[41]
CD4 binding	CD4-zeta chimeric T cell receptor	Syngeneic CD4^+^ and CD8^+^ T cells	0.1%–1% of CD4^+^ and CD8^+^ T cells at 1 year	[42]
CD4 binding	CD4-zeta chimeric T cell receptor	Autologous CD4^+^ and CD8^+^ T cells	0.1%–10% of PBMCs at 24 weeks	[43]
CCR5 binding	shRNA targeting tat/rev, TAR decoy, CCR5 ribozyme	Autologous CD34^+^ hematopoietic stem cells	0.01% of whole blood at 18 months	[44]
CCR5 binding	CCR5-specific Zinc Finger Nuclease	Autologous CD4^+^ T cells	Completed	[45]
CCR5 binding	CCR5-specific Zinc Finger Nuclease (dose escalation)	Autologous CD4^+^ T cells	Ongoing	[46]
CCR5 binding	CCR5-specific Zinc Finger Nuclease (dose escalation, with cyclophosphamide)	Autologous CD4^+^ T cells	Ongoing	[47]
CCR5 binding	CCR5-specific Zinc Finger Nuclease	Autologous hematopoietic stem cells	Ongoing	[48]
HIV fusion	HIV fusion inhibitory peptide maC46	Autologous CD4^+^ T cells	Less than 0.01% of leukocytes after day 7	[49]

## 4. HIV Entry as a Target for Gene Therapy

HIV entry is a multi-step process that begins with engagement of the host protein CD4 by the viral glycoprotein, Env. Env exists as a trimer of heterodimers on the surface of virions, and the Env heterodimer is made up of two subunits—the receptor and coreceptor binding subunit gp120, and gp41 which mediates membrane fusion. CD4 serves as the primary entry receptor for HIV [50,51] and binding of the gp120 subunit of Env to CD4 results in conformational changes in the viral glycoprotein that allow it to subsequently bind one of two coreceptors—either CCR5 or CXCR4 [52,53,54,55,56,57,58]. These coreceptors belong to a family of molecules called chemokine receptors, with important roles in immune cell signaling and trafficking. CCR5 is the primary coreceptor used in HIV-1 transmission [59,60,61,62] and its importance in establishing infection is further highlighted by the HIV-resistant phenotype of *ccr5*∆32 homozygotes. While not commonly used by transmitted viruses, CXCR4 also plays an important role in HIV pathogenesis as up to half of late-stage infected individuals harbor viruses capable of using CXCR4 to enter cells, and the presence of these viruses is associated with a more rapid progression to AIDS [63,64,65]. Coreceptor binding leads to exposure of the hydrophobic viral fusion peptide at the amino terminus of the gp41 subunit that subsequently inserts into the host cell membrane. Upon folding into a six-helix bundle involving all three gp41 subunits in an Env trimer, both membranes are brought into close enough apposition for membrane fusion to occur [66,67,68,69]. To date, several studies have explored genetic approaches to hijack or halt the 3 main steps of HIV entry—CD4 binding, coreceptor binding and membrane fusion.

Outside of its role as the primary receptor for HIV, CD4 plays a critical role in antigen recognition by the T-cell receptor and as such, abrogation of CD4 expression is not a viable option to prevent HIV entry. A number of early gene therapy studies, however, took advantage of the fact that HIV-1 requires CD4 to enter cells, and coupled the extracellular and transmembrane domains of CD4 to the intracellular signaling domain of the invariant ζ-chain of the T cell receptor (TCR) thus pairing viral recognition by CD4 with TCR signaling and downstream effector functions. Introduction of these chimeric receptors into CD4 and CD8 T cells resulted in HIV-specific targeting by both cell types *in vitro*. In particular, expression of these chimeric TCRs in cytotoxic CD8 cells allowed them to recognize and kill HIV infected cells [70,71]*.* Following these promising preclinical studies, two clinical trials investigated the effects of adoptive transfer of chimeric TCR modified CD4 and CD8 T cells on HIV infection. In both trials, the gene-modified cells successfully engrafted and trafficked to the rectal mucosa—a major site of HIV replication [41,42,43]. While neither study observed a significant decrease in the viral load of treated subjects, one study reported a trend towards a decrease in reservoir size following treatment with the gene-modified cells [43].

To date, most gene therapy attempts to inhibit HIV entry have focused on interfering with the interaction between the virus and its coreceptors by either reducing or eliminating coreceptor expression. When attempting to genetically modulate coreceptor expression, an important consideration is the choice of cells to be treated as the ease with which target cells can be modified, and their longevity and capacity for self-renewal all influence the chances of success (reviewed in [72]). For this reason, most studies have focused on modifying either T cells or CD34^+^ HSCs with the eventual goal of adoptive transfer of the gene-modified cells. CCR5 is a particularly attractive target for HIV entry-focused gene therapy as the complete loss of CCR5 expression appears to be well tolerated in *ccr5*∆32 homozygotes. Additionally, the CCR5 small molecule antagonist *maraviroc*, which is currently approved for use in HIV infected patients, exhibits potent antiviral activity without adversely affecting immune cell function, suggesting that a partial or complete loss of CCR5 may not result in severe immunologic consequences [38,39]. On the other hand, less is known about the potential consequences of decreasing or completely ablating CXCR4 expression. In particular, CXCR4 plays a key role in the bone-marrow retention of HSCs [73], and as such, permanent ablation of CXCR4 in these cells may result in the unintended side effect of HSC egress from the bone marrow into the peripheral blood.

A number of studies have demonstrated the ability of exogenous transgenes to target the HIV coreceptors at the level of protein, RNA or DNA, with the final common result being decreased surface coreceptor expression. Early work using a CCR5-specific single-chain antibody engineered to express an ER-retention motif showed that these “intrabodies” prevented trafficking of the CCR5 protein to the cell surface. When introduced into susceptible cells, the resulting intrabody-mediated intracellular sequestration of CCR5 resulted in a decrease in infection by CCR5-using HIV [74,75]. RNA-based approaches have also provided promising results—multiple studies have used RNA interference, CCR5-targeted ribozymes or a combination of both to efficiently decrease levels of CCR5 mRNA and thus surface expression of CCR5 [76,77,78,79,80,81,82]. A recent clinical trial examined the safety and potential efficacy of one such RNA-based agent by following the adoptive transfer of autologous CD34^+^ stem cells transduced with a lentivirus encoding a CCR5 ribozyme, an anti-HIV siRNA and an RNA decoy that prevents initiation of HIV transcription [44]. In this study, the gene-marked cells engrafted successfully, and multiple hematopoietic lineages expressing the transgene were detectable for up to two years post-infusion. However, these cells did not provide any observable clinical benefit in terms of CD4 count or HIV viral load. While the lack of clinical benefit was likely in part due to the low percentage of gene-marked cells infused (~0.14% of infused cells were gene-marked on average), another potential problem with this and other RNA-based gene therapy approaches to decrease CCR5 expression is their inability to completely and permanently eliminate surface CCR5 expression. This poses a problem, as many HIV-1 isolates are capable of using low surface levels of CCR5 to enter cells. For this reason, many groups have begun exploring permanent modification of the host genome using designer nucleases so as to completely eliminate surface CCR5 expression. 

Designer nucleases are a diverse family of chimeric proteins that can be engineered to bind any given DNA sequence and once at their target, introduce a double-strand break (DSB). Repair of the DSB occurs either by the error-prone non-homologous end-joining pathway, or the higher fidelity homologous recombination pathway. DSB repair by non-homologous end-joining results in random insertions and deletions that result in a non-functional gene product [83], and thus provide a way to permanently inactivate a given genomic target. As transient expression of a designer nuclease results in permanent genomic modification, the use of designer nucleases averts any potential insertional mutagenesis associated with retroviral vectors, and the potential immunogenicity that may result from permanent, stable expression of a foreign transgene. Of the three main designer nucleases—Zinc finger nucleases (ZFNs), Transcriptional Activator-Like Effector Nucleases (TALENs), and RNA-guided endonucleases (CRISPR/Cas9 systems)—ZFNs were the first designer nucleases used to modify the CCR5 locus [84]. ZFNs are chimeric proteins that function as a pair, and each member of the pair consists of a DNA-binding zinc finger protein fused to the nuclease domain of the FokI endonuclease [85,86,87]. Members of a ZFN pair bind opposite strands of DNA, and a DSB only occurs if the bound members of a ZFN pair are within 5 to 6 base pairs of each other. These strict binding criteria increase genome-wide specificity by decreasing the chances of unwanted binding and off-target genomic modification. CCR5-specific ZFNs are capable of permanently inactivating the CCR5 gene in primary CD4 T cells, and ZFN-modification confers a survival advantage on gene-modified cells in the presence of CCR5-using HIV both *in vitro* and in a humanized mouse model of HIV infection [84]. Additionally, ZFN modification of CCR5 in primary CD34^+^ HSCs results in the production of multiple hematopoietic lineages all lacking surface CCR5 expression [88]. As a result of these promising preclinical data, several clinical trials are currently investigating the safety and efficacy of autologous transplants using ZFN-modified CD4 T cells or CD34^+^ HSCs in HIV infected individuals [45,46,47,48]. While these strategies are capable of generating cells that are highly resistant to infection with CCR5-using HIV, they offer no protection against viruses that use CXCR4. To address this issue, a recent study took advantage of the fact that in the presence of a template with homology to the site of a DSB, repair of the break often occurs via homologous recombination. As such, they introduced a DSB using a CCR5-specific ZFN, and at the same time delivered a donor transgene with homology to CCR5 on either end, to simultaneously inactivate *ccr5* and insert a transgene encoding three HIV restriction factors—the dominant negative Rev M10 protein, the cytidine deaminase APOBEC3G, and the antiviral protein TRIM5α. This “stacking” of restriction factors led to a dramatic inhibition of infection by both CCR5 and CXCR4-using viruses [89]. While the designer nuclease approach remains a promising one, emerging evidence suggests that designer nucleases can cleave at unwanted or off-target sites. By searching the genome for sites with sequence similarity to the nuclease target site and analyzing these potential off-target sites for evidence of nuclease activity, several studies have identified some sites of off-target activity [84,90,91,92]. However, the *in vivo* relevance of these off-target effects remains to be seen. 

The final step in HIV entry—membrane fusion—represents another potential target for gene therapy approaches to block HIV infection. The HIV glycoprotein gp41 is responsible for membrane fusion, and contains two heptad repeat domains downstream of the *N*-terminal fusion peptide. Following insertion of the fusion peptide into the host cell membrane, the *N*- and *C*-terminal heptad repeats interact by folding back on each other to form a six-helix bundle. A number of peptide inhibitors with homology to these repeats interfere with the formation of the six-helix bundle, effectively inhibiting HIV-entry at the fusion step and one such peptide inhibitor, *enfuvirtide* (T20), is approved for use by the U.S. Food and Drug administration (Silver Spring, MD, USA). Another gp41 mimetic, C46, also inhibits entry at the fusion step, and a membrane anchored form of this peptide, maC46, prevents infection of cells expressing this peptide [93]. Following these promising preclinical data, a small clinical trial looked at the ability of CD4 T cells transduced with a retroviral vector expressing maC46 to engraft, persist, and modulate HIV infection in seven patients who harbored multi-drug resistant viruses [49]. While infusion of the gene marked cells was well-tolerated and the cells could be detected in multiple compartments up to a year post-infusion, there was no direct effect of this therapy on CD4 counts or viral loads, again likely due to low levels of gene-marking. However, in a recent study of the autologous transfer of maC46 treated HSCs in non-human primates, up to half of the infused cells were modified to express the maC46 construct, and under these conditions, durable effects on viral load and positive selection of gene-marked cells were observed [94], emphasizing the importance of achieving high levels of gene-marking in gene therapy approaches for HIV.

## 5. Graft *vs.* HIV: Allogeneic Stem Cell Transplantation and HIV Infection

Allogeneic HSCT has long been known to exert an anti-tumor or graft *vs.* tumor effect [95]. While there is conflicting evidence on the effect of allogeneic HSCT on HIV infection (reviewed in [96,97]), the idea that complete donor chimerism, if achieved following an allogeneic HSCT, would effectively cure an established HIV infection is an interesting one. For this to occur, the donor cells would likely need to be protected from infection during the transition to complete donor chimerism, and in the case of the *ccr5*∆32 transplant that resulted in a cure, that was achieved by using cells resistant to HIV infection. Another instance where protection of newly infused donor cells may have provided a clinical benefit in HIV infection is in the case of the “Boston patients”. In this small study, three HIV-positive patients in Boston received allogeneic HSCT following reduced-intensity chemotherapy to treat lymphoma. The patients were kept on ART for up to 4 years following transplant and while only two patients survived, they were both shown to have a significantly lower viral reservoir as measured by viral outgrowth following maximum *in vitro* T cell activation [40]. Moreover, these patients have recently undergone ART interruption and showed a delay in viral rebound of up to 32 weeks [98]. In this study, the cells used in the HSCT did not lack CCR5, and the procedure was performed with a reduced intensity chemotherapy regimen suggesting that this approach—if eventually successful—may sidestep some of the issues of HSCT toxicity related to chemotherapy, and the relatively low frequency of *ccr5∆32* homozygous donors that has plagued attempts to repeat the success of the first cure. Of note, the patient who received a transplant using *ccr5*∆32 cells and both Boston patients who survived allogeneic HSCT with *ccr5* wild-type cells were all *ccr5*∆32 heterozygotes at baseline. At this time, however, it is not known whether the *ccr5*∆32 heterozygosity or the use of ART in the post-transplant period [99] could have contributed to the delayed viral rebound seen in the Boston patients, or even a less severe course of GVHD in all three patients. 

## 6. Conclusions

Our thorough understanding of the biology of HIV infection comes from its place as one of the most intensely studied pathogens in recent history, and this knowledge has contributed to the many different strategies outlined here that are currently being tested for their ability to cure HIV infections. Each of these approaches—gene therapy, virus reactivation strategies, and allogeneic HSCTs—takes a slightly different approach in addressing the issue of a cure, and is faced with its own unique set of challenges. Efforts to reactivate the HIV reservoir must be coupled with ways of effectively killing reactivated cells harboring these reservoirs, gene therapy approaches must overcome issues of safety, appropriate choice of cell type and achieving levels of gene-marked cells capable of exerting a therapeutic effect, and the exact effect of HSCT on HIV infection remains unclear. However, the recent reports of a cure following *ccr5*∆32 HSCT, and the reductions in reservoir size observed in the Boston patients continue to reinforce the idea that while we are not there yet, a cure may still be just around the corner.
